# Designing, delivering and evaluating a specialty-specific quality improvement course for the rheumatology multidisciplinary team

**DOI:** 10.1093/rap/rkae110

**Published:** 2024-09-06

**Authors:** Rosalind M Benson, Charlotte A Sharp, Elizabeth M MacPhie, Hannah S Baird

**Affiliations:** Rheumatology, Aintree Hospital, Liverpool University Hospitals Foundation Trust, Liverpool, UK; The Kellgren Centre for Rheumatology, Manchester Royal Infirmary, Manchester University NHS Foundation Trust, Manchester, UK; Division of Musculoskeletal and Dermatological Sciences, School of Biological Sciences, Faculty of Biology, Medicine and Health, The University of Manchester, Manchester, UK; Rheumatology, Lancashire and South Cumbria NHS Foundation Trust, Preston, UK; Lancashire and South Cumbria Integrated Care Board, Preston, UK; Emergency Department, Royal Bolton Hospital, Bolton, United Kingdom

**Keywords:** quality improvement, education, training, medical education, experiential learning, rheumatology, evaluation

## Abstract

**Objectives:**

Quality improvement (QI) methodology aims to drive improvement in healthcare using a systematic approach. QI is an integral part of healthcare professional training curricula. However, many members of the rheumatology community have not accessed formal QI methodology training, including those expected to supervise QI activity. The BSR QI practical methodology workshop was created to address this knowledge gap in a specialty-specific course designed and delivered by, and for, the rheumatology multidisciplinary team.

**Methods:**

Course design centred on the Institute for Healthcare Improvement approach, ‘Model for improvement’, adapting materials from the well-established Trainees Improving Patient Safety through QI (TIPSQI) initiative. The course was delivered online (2021) and face-to-face (2022). Kolbs’ four-stage experiential learning cycle informed course design utilizing rheumatology-specific cases and facilitated breakout rooms to teach QI tools. Kirkpatrick’s four-stage model was used to design the course evaluation. Data from surveys completed before, immediately after, and 6 months following the courses, were used to evaluate the course.

**Results:**

Baseline knowledge of specific QI tools was limited. Post course evaluation demonstrated increased confidence to use and teach tools. Sustained confidence to contribute to and lead QI activity was reported. Course satisfaction was high; 100% of delegates would recommend the course to colleagues.

**Conclusion:**

This successful, rheumatology-specific QI course led to improved delegate knowledge of QI methodology and confidence in leading and teaching QI initiatives. It has contributed to building momentum in a growing rheumatology QI community of practice and to embedding a sustainable culture of improvement across the rheumatology community.

Key messages

- A QI course delivering formal QI training was developed to address a gap in knowledge identified amongst rheumatology professionals
- Course materials and structure can be adapted to suit other nations and specialties
- Workshops focused on building learning and networks across the MDT, creating a QI community of practice

## Introduction

Quality improvement (QI) methodology provides a systematic approach to plan and evaluate the impact of interventions aiming to make improvements in complex systems such as healthcare. Influential organizations worldwide have highlighted the role QI should play in driving improvement utilizing national audit data, including through the UK’s national early inflammatory arthritis audit [1, 2]. Increasing emphasis is placed upon all members of the multi-disciplinary team (MDT), including members of the rheumatology community, to undertake QI as part of professional practice [3].

The most commonly used methodologies for QI include, but are not limited to: Lean Methodology, Six Sigma and the ‘Model for Improvement’ (MFI) [4]. The MFI is structured around three key questions: What are we trying to accomplish? How will we know that a change is an improvement? What change can we make that will result in improvement? The MFI was developed by the Institute of Healthcare Improvement (IHI) in the USA. The IHI’s approach and the MFI have been adopted widely internationally [5, 6]. Common tools utilized to support the MFI include process mapping and driver diagrams. These tools enable users to develop change ideas and to test and evaluate improvement interventions in a systematic manner in iterative plan-do-study-act (PDSA) cycles.

There is increasing recognition that embedding QI skills within healthcare requires the building of capacity and capability within organizations [7]. This requires training of the MDT with sufficient understanding of QI principles that they can enable knowledge spread by teaching others. Educational theorists Lave and Wenger developed this concept further, defining ‘groups of people who share a concern or a passion for something they do and learn how to do it better as they interact regularly’ as a community of practice of like-minded individuals [8].

Arguably, the UK rheumatology community has had some catching up to do in QI compared both with other UK physicianly specialties [9] and the wider international rheumatology community [2]. The British Society for Rheumatology (BSR) first developed QI tools such as example driver diagrams to support the multi-regional SLE audit and national early inflammatory arthritis audits in 2018 and 2020 respectively [10–12]. However, developing tools as stand-alone interventions is unlikely to maximize the potential to improve patient care [1]. Instead, individuals need to be equipped, via training, with the skills to develop local QI initiatives, and to help capacity-build QI communities of practice in the organizations in which they are based [13].

In the UK, undertaking QI is mandatory for many trainees, and for consultant revalidation [14–16]. However, because QI has gained dominance in the UK only in the last 15–20 years, senior colleagues including consultants may not have had any formal training in QI approaches, leading them to feel under-confident in supporting trainees [17]. The availability and uptake of QI training varies across the UK. Given this context, the course conveners recognized a training need in QI methodology within the rheumatology community. The course aimed to enable learning and sharing of skills across the MDT, and to address the existing knowledge gap around QI methodology. The overall course objective was: ‘to improve QI capacity for the rheumatology MDT, by developing and delivering a QI course’.

## Methods

### Designing the course

Course content was based upon well-established resources developed by Trainees Improving Patient Safety through Quality Improvement (TIPSQI), a peer-led regional QI training programme [18]. Time was spent introducing the faculty and learning objectives (Supplementary Table S1, available at *Rheumatology Advances in Practice* online) before moving onto specific QI tools and techniques. The course was structured around three main topic areas (Table 1). Scaffolded learning was used to help the learner develop greater understanding of a new concept by presenting it in a variety of contexts [19]. For example, a combination of techniques were used to reinforce key messages from each topic area. Each topic was first introduced using instructional lecture-style teaching and an illustrative case study, followed by group discussion in breakout rooms. The first session focused on understanding the problem including how to: choose a project; process map; develop SMART aims; and use the MFI. The second session focused on techniques to measure variation and present data, followed by how to design and test interventions using Plan-Do-Study-Act cycles. The third session taught how to identify and engage stakeholders in QI activity. The inclusion of an expert patient in each course enabled the sharing of valuable insight, highlighting the need for inclusion of patients as stakeholders in project design. Patient contributors attending for the section on patient and public involvement and engagement were remunerated for their time and expert contribution.

**Table 1.** Overview of course structure 2021

Topic 1 – Understanding the problem	Introduction to improvement science and systems thinking
	What is QI
	How to choose a project
	Process mapping
	Model for improvement
	Developing a SMART aim
Topic 2 – Measurement for improvement	Measuring variation
	Run charts
	Change ideas
	PDSA cycles
Topic 3 – Engaging stakeholders	Stakeholder engagement
	Patient and public involvement.

**Figure 1.**
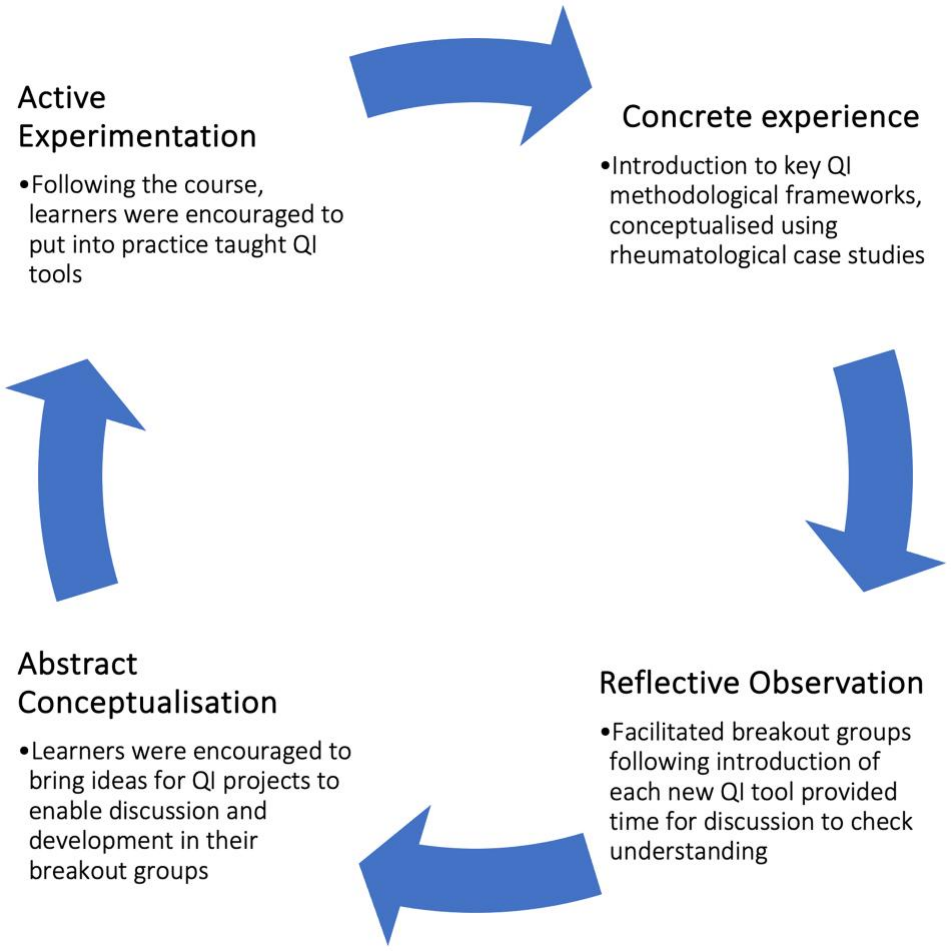
Kolb’s experiential learning cycle [21] illustrated to demonstrate how the principles were applied to course design

Course design was informed by feedback from a regional rheumatology QI training course [20] and a pre-course survey of participants. The course was designed by experienced QI practitioners in the specialty and was informed by the principles of Kolbs’ four-stage experiential learning cycle [21] (Fig. 1). During the first stage, ‘concrete experience’, the learner assimilates information by observing, being taught, or undertaking the activity. The instructional lecture-based sessions taught key QI methodologies and reinforced these using QI case studies. The second stage, ‘reflective observation’, occurs when the learner considers and reflects on their experience. The breakout rooms were used to enable such reflection and discussion. The third stage, ‘abstract conceptualization’ is when the learner forms new ideas or modifies existing ideas based on their previous reflections. Again, the breakout groups gave participants the chance to revise their existing plans for QI projects. The final stage, ‘active experimentation’ occurs when the learner tries out what they have learned. Participants were encouraged to utilize the QI tools and techniques in practice following the course.

Montgomery *et al.* [22] advocate the construct of ‘team capital’ in relation to QI work. First developed by Bourdieu, this construct describes the ‘key driving mechanism for establishing, maintaining and reproducing social relationships within and across different fields of practice’ [23]. Qualitative analysis of QI projects utilizing MDTs of varying seniority found those with greater ‘team capital’ yielded more ambitious projects with greater progress charted. Aiming to replicate this within the course structure, course convenors drew on many skilled QI practitioners from across the MDT, including, for example, physiotherapists teaching consultants in an interaction that is not necessarily routine in the rheumatology community. In order to build capacity in facilitating QI training, the course faculty included different personnel in 2021 and 2022.

### Delivering the course

QI principles are strongly underpinned by engagement with the MDT and patients [24] and collaborative working is integral to the development of a thriving community of practice [8]. Course delegates held varying experience in QI, including some novices. Legitimate peripheral participation is based on Lave and Wenger’s theory of situational learning [25]. It describes the process of a novice initially seated at the periphery of the community of practice gradually moving towards the centre as they choose to engage, interact and collaborate further with those who are more ‘expert’. Each breakout group comprised a facilitator and four/five delegates who were selected based on the group’s project topics, individual professional roles and degree of experience in QI. This enabled multi-disciplinary networking and provided peer support for related projects with the aim of facilitating legitimate peripheral participation. A pre-course survey helped facilitators understand their groups’ QI knowledge and subsequently pitch their facilitation accordingly.

The first BSR QI practical methodology workshop was delivered virtually in 2021 using a virtual platform, BigBlueButton™, during a period of ongoing uncertainty regarding COVID-19 restrictions. The 2022 course was held face to face following easing of those restrictions. Pre-course supportive materials were available to delegates 2 weeks prior to the course. Key opinion leaders set the scene for the day, highlighting the policy context and importance of QI for the rheumatology community. Content for each topic was delivered by the core team (R.B., C.A.S., H.B.) using PowerPoint presentations, followed by an opportunity to reflect on and apply the tools in breakout groups.

### Evaluating the course

**Figure 2.**
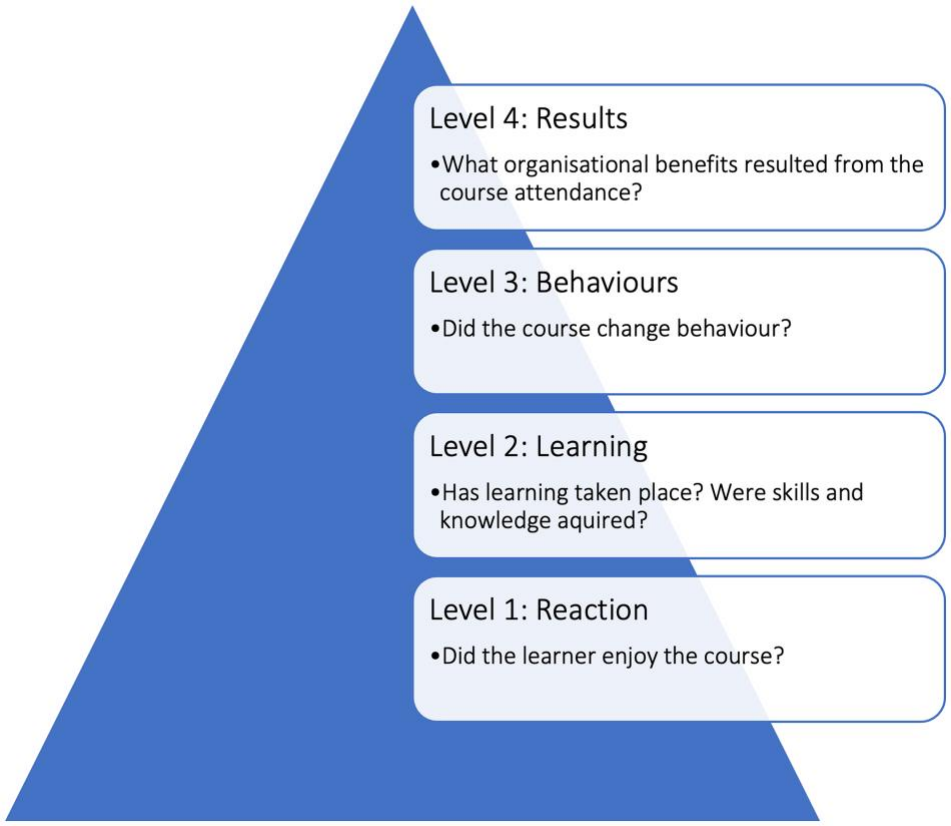
Kirkpatrick Evaluation model [26] illustrated to demonstrate evaluation of the QI course

Kirkpatrick’s four-level evaluation model was designed to evaluate the effectiveness of teaching interventions [26]. We illustrated the model to help structure the evaluation of the QI course (Fig. 2). The first stage relates to a learner’s enjoyment of a course. The second stage addresses whether learning has taken place. The third stage questions whether the course changed the learner’s behaviour. The fourth stage asks what organizational benefits have resulted from learners attending the course. Detailed written feedback was collected from course delegates immediately following the course, and six months later. Responses were compared with the pre-course survey results and analysed using descriptive statistics.

**Figure 3.**
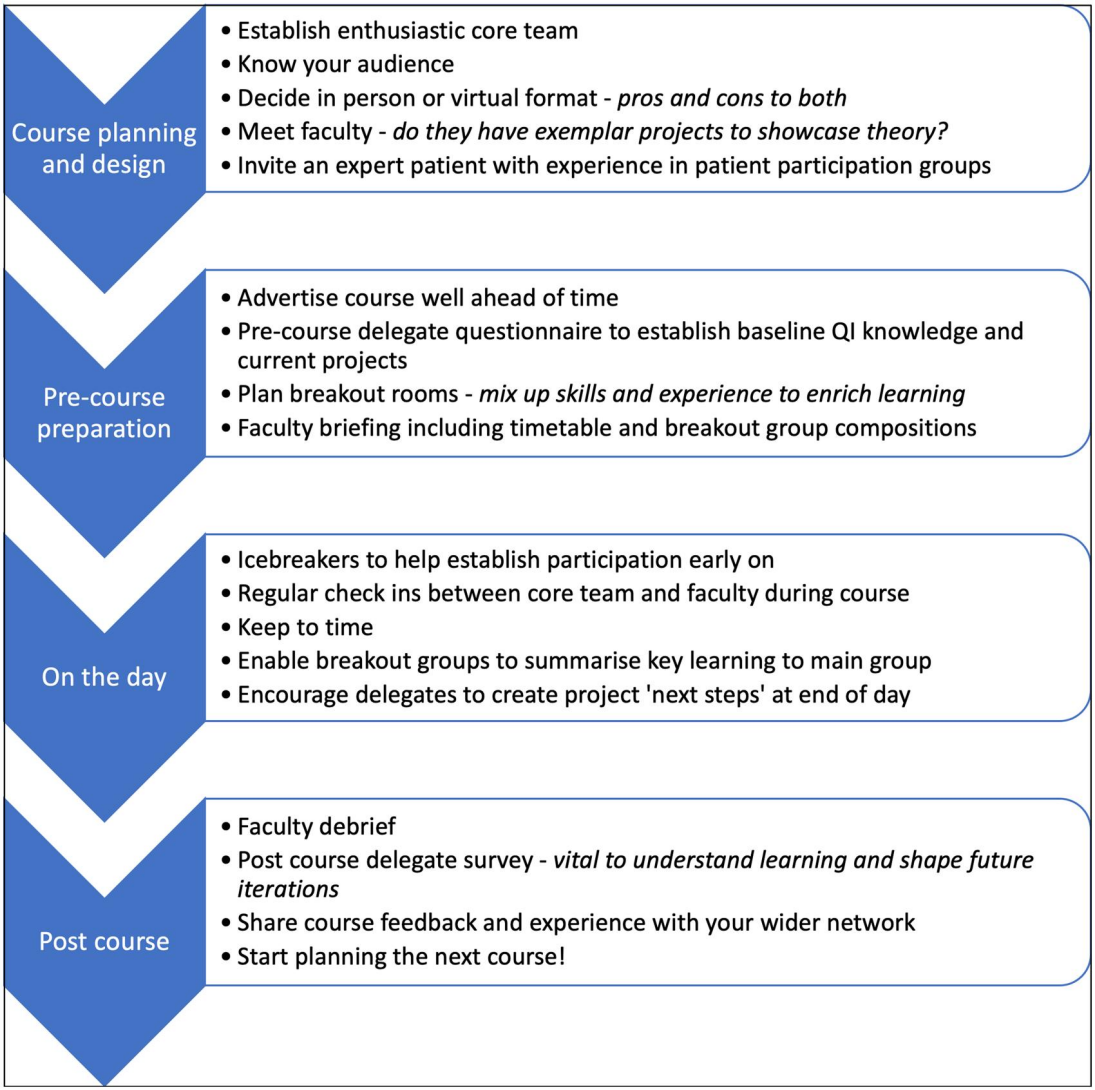
Top tips for developing your own quality improvement course

Feedback from 2021 informed course design for 2022. More time was allotted to breakout groups to enable exploration of projects with facilitators, increasing the opportunity for the second and third stages of Kolb’s learning cycle, reflective observation and abstract conceptualization. Stakeholder engagement was moved up the agenda to highlight the importance of establishing positive relationships early on in projects. Greater emphasis was placed on the overarching structure of the MFI, and progress through the model was highlighted in each course section. In 2021, delegates reported gaining less confidence in involving patients than in other areas of the course. Consequently, the patient contribution in 2022 was adjusted to include a more practical focus on top tips for patient collaboration. Using the learning gained from running the courses, we developed a top tips sheet for developing your own QI course (Fig. 3), which may be adapted and applied to a range of settings. This is not a research study and therefore ethical approval was not required.

## Results

Delegates from both years included consultant and trainee doctors, pharmacists, nurses and physiotherapists from all four UK nations. Thirty people attended in 2021, with 28, 22 and 4 completing pre-course, post-course and six-month surveys, respectively. Twenty-three people attended in 2022, with 23, 22 and 9 completing pre-course, post-course and six-month surveys, respectively. Full results are presented in Supplementary Tables S1 (2021) and S2 (2022), available at *Rheumatology Advances in Practice* online.

For Kirkpatrick level 1, ‘reaction’, respondents reported high levels of satisfaction. One hundred percent of respondents across both years stated that they would recommend the course to colleagues. Mean levels of satisfaction were 4.5 and 4.9 out of 5 for 2021 and 2022, respectively. The latter was the highest level of satisfaction reported for a BSR course. A large number of positive free text comments were received for both years.

Driver diagrams can be used as an exemplar to evaluate Kirkpatrick level 2: ‘learning’. Before the courses, 57% (2021) and 43% (2022) did not know what a driver diagram was. Before the 2021 course, 10.1% felt confident to use a driver diagram, with no delegates prior to 2022 feeling confident to use one. Following both courses, 100% of delegates felt confident developing them, an improvement which persisted at 6 months. No delegates felt confident teaching driver diagrams prior to the courses. Following the 2021 course, 54.6% felt confident to teach their use, a change which persisted at 6 months. In 2022, 35% felt confident to teach the use of driver diagrams, although this dropped off to nil by 6 months. Similar findings were identified across all the tools over both years, with the biggest improvements occurring for driver diagrams in 2021 and run charts in 2022. Confidence in contributing to QI, leading QI and teaching QI improved after both courses. Confidence rose again a further six months after both courses. The biggest increase in 2021 occurred around confidence in contributing to a QI projects, with a positive change of 2.2 Likert points. In 2022 confidence in engaging patients in projects also increased by 2.2 Likert points.

Evaluating Kirkpatrick level 3, ‘behaviour’, 100% of respondents from both years reported that the course would change their way of practicing QI. All four 2021 respondents to the 6 month survey and 55% of the nine respondents to the 2022 6 month survey had performed QI following the course. Two respondents from each year had conducted teaching on QI, representing 50% of respondents for 2021 and 22% from 2022.

In evaluating Kirkpatrick level 4, ‘results’, 1/4 (25%) of 2021 respondents felt the course had changed the impact of their work. Three/four (75%) reported time and institutional constraints as barriers to conducting QI. Delegates attending the 2022 course reported increased impact of their QI projects as a consequence of attending the course in 66% of the nine responses received. Reported barriers to conducting QI repeated those identified in 2021. They included a lack of engagement from managers, colleague inertia, staff turnover and difficulties obtaining data.

## Discussion

### Course impact

Building on TIPSQI’s established and successful course framework meant we could focus on tailoring content to our specific rheumatology community rather than having to design all the content anew [18]. These resources remain open-access and could be used and adapted for other settings internationally. The demonstration of QI tools in the context of real world rheumatology QI projects by members of the MDT enabled delegates to deepen their understanding, applying the principles of scaffolded learning [19]. The benefits of this approach were demonstrated clearly when comparing pre and post course surveys. Improvement in knowledge, confidence to use and teach were shown in relation to all QI tools discussed (Kirkpatrick level 2).

Demonstrating change in behaviour subject to an educational intervention (Kirkpatrick level 3) can be difficult; it is often reliant upon self-assessment and may not take account of environmental challenges [27]. Encouragingly however, a majority of course delegates stated that the course had influenced the impact of their QI projects on practice for the better, providing evidence for its efficacy. Confidence in contributing, leading and teaching QI increased before and after the course across both years. That it also increased following the courses suggested that work in practice had cemented the learning undertaken during the course. These findings demonstrate the value of bespoke QI courses aimed at specific communities such as rheumatology.

In line with the literature, delegates found that QI projects were often reliant on the enthusiasm of individuals. Barriers to improvement included a lack of time, colleague inertia and institutional constraints, which highlights the importance of systems approaches to improvement [28, 29]. Post course evaluation demonstrated that for each QI tool taught >40% of delegates were confident in teaching these tools to others. By 6 months however, the confidence to teach others certain tools including process mapping and run charts had decreased. This reflects the need for ongoing knowledge building to sustain confidence particularly in relation to capacity building. Future educational offerings for QI within the UK rheumatology community should pay attention to more supported longitudinal learning, to help delegates navigate these institutional barriers.

Evaluation of the longer-term impact of the course is limited by the smaller numbers of survey data and relatively short time frame (6 months) between the course and final surveys. Caution in interpreting these data should consider the low response rate for 6-month surveys (2021) and recognition that QI projects can often take more than six months from conception to completion, longer than the timeframe for the surveys.

### Developing a community of practice

The QI course was designed and delivered by experienced trainees, teaching up to consultants and other senior colleagues, in response to the situation that has evolved where senior colleagues may not feel confident in supervising projects [17]. Facilitators from across the MDT reinforced networking opportunities and idea sharing. Benefits included sharing ideas and the opportunity for delegates to have protected time to develop their own projects with the support of peers attending the course. In the course feedback, many delegates commented on the richness of the learning experience delivered within the breakout group environment. Feedback provided evidence that the carefully constructed breakout groups in particular, enabled legitimate peripheral participation as novices became more expert and involved in QI [25]. The course was seen as an opportunity to engage with a growing QI community of practice within the rheumatology community [25], leading participants to teach and lead QI.

## Conclusion

The BSR practical methodology QI workshops were designed and delivered by trainees, teaching up to consultants and other senior colleagues. A combination of instructional lectures, case based examples and breakout group work reinforced key messages. Use of well-established training materials demonstrates that the course content can be adapted for other contexts. Feedback demonstrated improved delegate knowledge of QI processes and tools, stakeholder engagement and patient and public involvement in improvement. Course evaluation demonstrated that delegates felt more confident after the course in using the tools themselves, in leading QI initiatives, and in teaching others key QI tools and techniques.

Embedding educational theory into the course design supported successful cascading of knowledge to delegates. The course was held successfully both in-person and virtually, demonstrating flexibility and enabling future iterations of the course to take place in either environment. Both the structure and content of the course could be reproduced in other settings including different countries and different specialties, tailored to specific local contexts.

With skilled course faculty representation from experts including patients and the breadth of the MDT, the course builds on the ongoing development of a community of practice within the rheumatology specialty. The course will help develop a sound theoretical grounding as enthusiasm for QI within the rheumatology community grows. The challenge remains for the rheumatology community to embrace the momentum from recent years to continue to build expertise in QI and embed a sustainable culture of improvement internationally.

## Supplementary Material

rkae110_Supplementary_Data

## Data Availability

Survey data are held by British Society for Rheumatology and cannot be shared.
