# Supplementary material for: Designing, delivering and evaluating a specialty-specific quality improvement course for the rheumatology multidisciplinary team
Source: Rheumatol Adv Pract. 2024 Sep 6;9(2):rkae110. doi: 10.1093/rap/rkae110 (PMC11908373; doi:10.1093/rap/rkae110)
Supplement: rkae110_Supplementary_Data [file rkae110_supplementary_data.docx]

**Supplementary Table S1:** Pre-course, post-course, and six-month survey results from 2021

|  | Pre-course N = 28 | Post-course N=22 | Six-month N=4 |
| --- | --- | --- | --- |
| KIRKPATRICK LEVEL ONE: REACTION | | | |
| Would you recommend this course to a colleague? | N/A | Yes: 22/22 (100%) | N/A |
| Please indicate your overall satisfaction with the course?  1-not at all satisfied,  5-very satisfied | N/A | MEAN 4.5 | N/A |
| KIRKPATRICK LEVEL TWO: LEARNING | | | |
| Driver diagrams | | | |
| Do you know what a driver diagram is? | No: 16/28 (57.2%)  Yes: 9/28 (32.1%)  Unsure: 3/28 (10.7%) | N/A | N/A |
| Do you feel confident to use a driver diagram? | Yes: 3/28 (10.1%) | Yes: 22/22 (100%) | Yes: 4/4 (100%) |
| Do you feel confident to teach someone to use a driver diagram? | Yes: 0 (0%) | Yes: 12/22 (54.6%) | Yes: 2/4 (50%) |
| Process mapping | | | |
| Do you know what process mapping is? | Yes: 12/28 (43%) | N/A | N/A |
| Do you feel confident to process map? | Yes: 7/28 (25%) | Yes: 22/22 (100%) | Yes: 3/4 (75%) |
| Do you feel confident to teach someone to process map? | Yes: 0/28 (0%) | Yes: 10/22 (47.6%) | Yes: 0/4 (0%) |
| PDSA (plan-do-study-act) cycles | | | |
| Do you know what a PDSA cycle is? | No: 5/28 (17.8%)  Yes: 13/28 (46.1%)  Unsure: 10/28 (35.7%) | N/A | N/A |
| Do you feel confident to carry out a PDSA cycle? | Yes: 9/28 (32.1%) | Yes: 22/22 (100%) | Yes: 3/4 (75%) |
| Do you feel confident to teach someone to carry out a PDSA cycle? | Yes: 1/28 (3.6%) | 12/22 (54.6%) | Yes: 3/4 (75%) |
| Confidence in carrying QI projects | | | |
| How confident are you at contributing to a QI project?  1-not at all confident,  5-very confident | Mean 2.3 | Mean 4.36 | Mean 4.5 |
| How confident are you at leading a QI project?  1, not at all confident - 5, extremely confident | Mean 3.36 | Mean 3.95 | Mean 4.5 |
| How confident do you feel in engaging patients in projects?  1, not at all confident - 5, extremely confident | Mean 3.07 | Mean 3.72 | Mean 3.75 |
| KIRKPATRICK LEVEL THREE: BEHAVIOUR | | | |
| Will you change your way of practicing as a result of the course? | N/A | Yes: 22/22 (100%) | N/A |
| Have you done any QI work since the course? If so, how did the course influence that? | N/A | N/A | Yes: 4/4 (100%) |
|  |  |  | Comment:  *Confidence to use tools* |
| Have you taught or supervised anyone in QI since doing the course? If so, how did the course influence that? | - | - | Yes: 2/4 (50%) |
|  |  |  | Comment: *Provided a structure to support others doing QI* |
| KIRKPATRICK LEVEL FOUR: RESULTS | | | |
| Has attending the QI course changed the impact of your QI projects? If so, please explain how? | - | - | Yes: 1/4 (25%)  No: 3/4 (75%) |
|  |  |  | Comment: *Provided more structure* |
| Have you experienced any barriers to conducting QI in the way that you would like to? | - | - | Yes: 3/4 (75%) No: 1/4 (25%) |
|  |  |  | Comment: *Time and institutional support* |
| Free text comments | “*I think it was an excellent course for those starting on QI projects. It gives a structured direction to the project”*  *“Fantastic course to equip you with the tools and confidence to launch yourself into the world of QI. Just the right balance of theory and practical workshops.”*  *“I have always [been] interested in QI. This course has definitely enthused me further in contributing and leading QI, with better understanding in applying QI tools. Thank you!”* | | |

**Supplementary Table S2.** Pre-course, post-course, and six-month survey results from 2022

|  | Pre-course N = 22 | Post-course N=21 | Six-month survey N=9 |
| --- | --- | --- | --- |
| KIRKPATRICK LEVEL ONE: REACTION | | | |
| Would you recommend this course to a colleague? | N/A | Yes: 21/21: (100%) | N/A |
| Please indicate your overall satisfaction with the course?  1-not at all satisfied,  5-very satisfied | N/A | MEAN 4.9 | N/A |
| KIRKPATRICK LEVEL TWO: LEARNING | | | |
| Driver diagrams: How do you feel about driver diagrams? | | | |
| Do not know what driver diagrams are | 9/21 (43%) | 0 | 0 |
| Aware of driver diagrams but not sure how to use one | 12/21: (57%) | 0 | 0 |
| Aware of driver diagrams and confident developing one | 0 | 13/20: (65%) | 9/9: 100% |
| Aware of driver diagrams and confident to teach someone else to use one | 0 | 7/20: (35%) | 0 |
| Process mapping: How do you feel about process mapping? | | | |
| Do not know what process maps are | 7/21: (33%) | 0 | 0 |
| Aware of process maps but not sure how to use one | 10/21: (48%) | 0 | 1/9: (11%) |
| Aware of process maps and confident developing one | 4/21 (19%) | 11/20: (55%) | 7/9: (77%) |
| Aware of process maps and confident to teach someone else to use one | 0 | 9/20: (45%) | 1/9: (11%) |
| PDSA (plan-do-study-act) cycles: How do you feel about a PDSA (plan-do-study-act) cycle? | | | |
| Do not know what they are | 4/21: (19%) | 0 | 0 |
| Aware of them but not sure how to do one | 11/21: (52%) | 0 | 0 |
| Aware of them and confident performing one | 5/21: (24%) | 10/20: (50%) | 6/9: (66%) |
| Aware of them and confident to teach someone else to carry out a PDSA cycle | 1/21: (5%) | 10/20: (50%) | 3/9: (33%) |
| Run charts: How do you feel about run charts? | | | |
| Do not know what run charts are | 11/21: (52%) | 0 | 0 |
| Aware of run charts but not sure how to use one | 10/21: (48%) | 0 | 0 |
| Aware of run charts and confident developing one | 0 | 12/20: (60%) | 7/9: (77%) |
| Aware of run charts and confident to teach someone else to use one | 0 | 8/20: (40%) | 2/9: (22%) |
| Confidence in carrying out QI projects | | | |
| How confident are you at contributing to a QI project?  1-not at all confident,  5-very confident | MEAN 3.3 | MEAN 4.2 | MEAN 4.4 |
| How confident are you at leading a QI project?  1, not at all confident - 5, extremely confident | MEAN 2.9 | MEAN 4.2 | MEAN 4.4 |
| How confident do you feel in engaging patients in projects?  1, not at all confident - 5, extremely confident | MEAN 2.6 | MEAN 3.9 | MEAN 4.4 |
| KIRKPATRICK LEVEL THREE: BEHAVIOUR | | | |
| Will attending the course have an impact on the way in which you work? | N/A | Yes 21/21 (100%) | N/A |
|  |  | Comments: *Yes, I feel more prepared to lead and supervise on a QIP; I came with a project in mind, I now have a clear plan on how I am going to implement that; Given me lots of thoughts for how I teach QI concepts to my team; More confident in developing QI project* |  |
| Have you done any QI work since the course? If so, how did the course influence that? | N/A | N/A | Yes: 5/9 (55%)  No: 4/9 (44%) |
|  |  |  | Comments: *Gave tools and confidence to initiate. Gave basis and structure to develop it.* |
| Have you taught or supervised anyone in QI since doing the course? If so, how did the course influence that? | N/A | N/A | Yes: 2/9 (22%)  No: 7/9 (77%) |
|  |  |  |  |
| KIRKPATRICK LEVEL FOUR: RESULTS | | | |
| Has attending the QI course changed the impact of your QI projects? If so, please explain how? | N/A | N/A | Yes 6/9 (66%)  No 3/9 (33%) |
|  |  |  | Comments: *Achieved the improvement aimed at; I think the patient and public engagement makes the project more credible; Made it more practical* |
| Have you experienced any barriers to conducting QI in the way that you would like to? | N/A | N/A | Yes: 4/9 (44%)  No: 5/9 (55%) |
|  |  |  | Comments: *Inertia from colleagues in other department for change was the biggest barrier, Time constraint, Manpower shortage; lack of engagement from the managers; Institutional barriers remain - time in job plan, implementing change due to staff shortage, staff turnover, lack of other staff engagement; engaging team in data recruiting* |
| Free text comments | *Good course; Great overview; Excellent course; Explained so many confusing concepts* | | |
